# Supplementary material for: Academic enjoyment, behavioral engagement, self-concept, organizational strategy and achievement in EFL setting: A multiple mediation analysis
Source: PLoS One. 2022 Apr 29;17(4):e0267405. doi: 10.1371/journal.pone.0267405 (PMC9053798; doi:10.1371/journal.pone.0267405)
Supplement: Attachment — Submitted filename: PONE-D-21-30639.pdf.docx [file pone.0267405.s001.docx]

**Academic Enjoyment, Behavioral Engagement, Self-concept, Organizational Strategy and Achievement in EFL Setting: A Multiple Mediation Analysis**

This study aimed to investigate whether behavioural engagement, self-concept, and organizational strategy mediated relations between academic enjoyment and achievement in an English as a foreign language setting in China. Overall, 528 learners participated in the study. The paper has several strengths (e.g., a decent sample size and strong data analysis section). However, I believe there are a number of issues that need to be addressed before considering this paper for publication. Please see my comments below which are in no particular order of importance.

1. **Abstract:**

- The abstract needs to be improved.

a. The first two sentences need to be revised - the authors need to highlight the need for studies exploring the mechanisms through which enjoyment predicts academic achievement.
b. The abstract does not include all the information needed for the reader. E.g., there is no indication where this study took place and age/gender of the participants.
c. In the abstract, the authors just report the percentages of the mediating effects. I would suggest that they simplify this section and just directly state whether the proposed mediators mediated the relations between enjoyment and achievement.
2. **Introduction and literature review**

- In the introduction, the authors make false claims – e.g.,

“However, except for a few studies focusing on language anxiety, positive achievement emotions, for example, academic enjoyment, was seldom explored in the EFL setting”

a. Anxiety is not a positive emotion.
b. There are at least 10 studies conducted by Prof Jean-Marc Dewaele and many other recent publications by other scholars. In fact, language enjoyment is the most widely studied positive emotion in the field.

- I expected to read more information about why language enjoyment or positive emotions more generally matter for language achievement. There are loads of studies on emotions in the field – so rather than claiming that there are little research, I would suggest that the authors highlight the importance of the topic and why we need to explore the mechanisms through which enjoyment predicts language achievement.
- It is not clear anywhere in the manuscript how the authors decided on the mediators. Why did they choose “Behavioural Engagement, Self-concept, Organizational Strategy” as the mediators? Why was it important to explore these three constructs together?
- Did the authors use the CVT as their theoretical framework? Then, what does the CVT say about emotions predicting the achievement? They need to expand on this a bit more.
- The relations between the study variables should be better articulated. The authors say, for example,: “Goetz et al. (2008) examined the mediating effect of academic self-concept on mathematics-related achievement and enjoyment.” In this study, enjoyment seems to be the outcome and self-concept is the predictor – how do the authors explain enjoyment being predictor and self-concept the outcome/mediator in their study?
- There needs to be a section or at least mention of a few studies showing the relations between the mediators.

3. **Method & Results**

- In 2.3., the authors repeat that “However, studies have yet to investigate how positive achievement emotions (e.g., enjoyment) promote academic achievement in the EFL context”. Again, there are studies investigating these relations already. I suggest they highlight the strengths of their study and why it is needed/different/important.
- The section 3.1. needs to be revised. Some sections are not clear – e.g., students “…were told that they could choose not to fill out the questionnaire without affecting them”.
- Also, in Section 3.1., the authors need to explain the context a bit more. Currently, the only information we have about the participants is that they are secondary school students based in China. I think we need more information about their context to make better sense of why enjoyment matters for this particular group of learners.
- Measurement invariance should be reported before the mediation analyses are presented.
- Regarding the measurement invariance, I wonder why the authors did not test residual variance. They need to explain it.
- It is difficult to follow 4.3.2. I suggest the authors do not include the CIs in text as they are already presented in the table.

4. **Discussion and Conclusions**

- Discussion needs to be improved.

E.g. the authors state that: “With the rise of positive psychology, a growing number of studies, especially in STEM domains, have concentrated on how positive achievement emotions relate to the subsequent academic results.” This also applies to the field of SLA.

- The authors claim that “However, most of the existing studies only discussed one single mediator between these two constructs, and few studies have simultaneously investigated multiple mediators.” I think they need to provide the relevant citations here. Which studies do they refer to?
- The authors need to improve the implications section. Currently, the only suggestion they give is to increase enjoyment in the EFL classrooms. I think there are more implications to discuss here.

5. **Other issues:**

- Proofreading is needed.
- Citations and references need to be checked.
